# Persistent Pruritic Linear Streaks of Adult-Onset Still’s Disease: Reconsidering the Yamaguchi Criteria

**DOI:** 10.7759/cureus.62267

**Published:** 2024-06-12

**Authors:** Keiichi Iwanami, Takuya Hayase, Yohei Masuda, Atsushi Nomura, Yusuke Nakamichi, Eiji Hiraoka

**Affiliations:** 1 Department of Rheumatology, Tokyo Bay Urayasu Ichikawa Medical Center, Urayasu, JPN; 2 Department of Internal Medicine, Tokyo Bay Urayasu Ichikawa Medical Center, Urayasu, JPN; 3 Department of Rheumatology, Ushiku Aiwa General Hospital, Ushiku, JPN; 4 Department of Rheumatology, Tokyo Metropolitan Ohtsuka Hospita, Tokyo, JPN

**Keywords:** yamaguchi criteria, linear streaks, pruritic rash, diagnosis, atypical rash, adult-onset still’s disease

## Abstract

Objective

Adult-onset Still’s disease (AOSD) is a rare orphan disease, the diagnosis of which remains challenging. This study aimed to identify additional clues for establishing early diagnosis beyond the existing criteria.

Methods

A retrospective longitudinal cohort study was conducted at two community hospitals in Japan between March 2012 and December 2022. The clinical characteristics and medical histories of patients with AOSD were extracted from the clinical records. The primary outcome was to identify the key manifestations of AOSD for an early diagnosis beyond the existing criteria.

Results

Twenty-one patients (mean age, 58 years) were included in the study. Fever was the first symptom in 13 out of 21 patients (62%). Six out of 21 patients (29%) presented with a pruritic rash only, while two out of 21 (10%) initially presented with a sore throat. All patients visited more than one medical institution. The median time to reach a correct diagnosis was 41 days (IQR 19-138). Nineteen out of 20 patients (95%) exhibited a pruritic rash, identified as persistent pruritic linear streaks, with a median duration of 21 days (IQR 12-64) before the diagnosis of AOSD as a cutaneous manifestation.

Conclusions

Persistent pruritic linear streaks were a key feature in the context of an early diagnosis of AOSD, offering an option for reconsidering and revising the existing classification criteria.

## Introduction

Adult-onset Still’s disease (AOSD) is a rare systemic inflammatory disorder characterized by fever, arthralgia, and a distinctive salmon-pink evanescent rash. The diagnosis of AOSD is typically based on the Yamaguchi criteria, which comprise nonspecific manifestations and laboratory abnormalities. Among these features, a “typical rash” has the highest specificity (99%) and high sensitivity (87%) [1]. The requirements for a typical rash are as follows: (i) macular or maculopapular, (ii) nonpruritic, (iii) salmon-pink appearance, and (iv) fever and evanescence with defervescence. However, in clinical practice, a macular, maculopapular, or nonpruritic pink-colored eruption by itself (requirements (i)-(iii)) is nonspecific and frequently seen in drug and viral eruptions. The features of emergence and evanescence (requirement (iv)) are often overlooked by physicians, as they tend to consider a drug or viral eruption because both conditions can account for other clinical features of the Yamaguchi criteria such as fever, arthralgia, and laboratory abnormalities. This inclination may be a major cause of diagnostic delays in patients with AOSD. Since pruritic linear streaks resembling flagellate erythema can be associated with AOSD [2], this study aimed to explore their diagnostic value for AOSD in the context of an early diagnosis.

## Materials and methods

This was a retrospective study approved by the Institutional Ethics Committees of Tokyo Bay Urayasu Ichikawa Medical Center (approval number: 861) and Nerima Hikarigaoka Hospital (approval number: 23110901), Japan. The study followed the Strengthening the Reporting of Observational Studies in Epidemiology (STROBE) guidelines [3]. The requirement for written informed patient consent was waived by the Ethics Committees.

We retrospectively identified all patients diagnosed with AOSD based on the Yamaguchi criteria (Table 1) at the Tokyo Bay Urayasu Ichikawa Medical Center and Nerima Hikarigaoka Hospital, Japan, between March 2012 and December 2022. The diagnosis of AOSD was confirmed by experienced rheumatologists after excluding other potential causes of the patients’ clinical presentations such as infections, malignancies, and other rheumatic diseases.

**Table 1:** Yamaguchi criteria for adult-onset Still’s disease ^*^ Macular or maculopapular nonpruritic salmon-pink eruption usually appearing during fever; ^†^ Lymphadenopathy is defined as recent development of significant lymph node swelling, and splenomegaly is confirmed on palpation or by an echogram; ^‡^ Liver dysfunction is defined as an abnormally elevated level of transaminases and/or lactate dehydrogenase, which is attributed to liver damage associated with this disease but not with drug allergy/toxicity or other causes. For the differentiation, it is recommended to see if liver function returns to normal upon discontinuation of hepatotoxic drug or not, before applying this criterion; ^¶^ RF in serum must be negative by routine test for the detection of IgM RF, and serum ANA must be negative by routine immunofluorescence test All criteria are applicable only in the absence of other clinical explanations. RF: rheumatoid factor, ANA: antinuclear antibody.

Major criteria
1. Fever of 39℃ or higher, lasting one week or longer
2. Arthralgia lasting two weeks or longer
3. Typical rash^*^
4. Leukocytosis (10,000mm^3^ or greater) including 80% more of granulocytes
Minor criteria
1. Sore throat
2. Lymphadenopathy and/or splenomegaly^†^
3. Liver dysfunction^‡^
4. Negative rheumatoid factor and negative antinuclear antibody ^¶^
Exclusions
Ⅰ. Infections (especially, sepsis and infectious mononucleosis)
Ⅱ. Malignancies (especially, malignant lymphoma)
Ⅲ. Rheumatic diseases (especially, polyarteritis nodosa and rheumatoid vasculitis

The following clinical data were extracted to analyze the characteristics and clinical course: (i) initial symptoms, (ii) days from fever onset to the development of skin rash or vice versa, (iii) initial diagnosis at the first visit, and (iv) days from initial symptoms to diagnosis. All clinical manifestations were also reviewed, with a particular focus on salmon-colored evanescent rashes and persistent pruritic rash. Available clinical photographs of the skin rashes were also collected.

## Results

Twenty-one patients were identified. Fever was the first symptom in 13 out of 21 patients (62%). Regarding the initial symptoms of the remaining patients, six out of 21 patients (29%) presented only with a pruritic rash, and two out of 21 patients (10%) presented with a sore throat alone (Tables 2, 3).

**Table 2:** Characteristics of patients With AOSD ^†^ The salmon-pink rash may have developed before the days indicated in the table, as the non-pruritic nature of skin lesions could have led patients and physicians to overlook them; ^‡ ^The salmon-pink rash was always accompanied by fever; ^§ ^There was a possibility that a salmon-pink rash had developed prior to the patient’s visit to our hospital, but the patient’s memory was ambiguous; ^¶ ^There was a possibility that the pruritic rash and salmon-pink rash had developed at a previous hospital, but the date of emergence of salmon-pink rash and whether the pruritic rash developed were unclear in the record; ^**^ Some kind of skin rash associated with AOSD developed on day 17 (16 days after fever), but details were unavailable in the records from the previous hospital; ^*^ The order of appearance of the skin rash and fever was unknown. Additionally, the date of emergence of the pruritic rash and salmon-pink rash was unknown. N/A: not available; AOSD: adult-onset Still’s disease

Case (N=21)	Sex	Age Range (years)	Initial symptom	Days from fever to pruritic/salmon-pink rash (days)^†^	Days from pruritic rash to fever (days)^‡^	Salmon-pink evanescent rash	Persistent pruritic linear streaks
1	F	50s	Fever, Sore throat Arthralgia	8/21	-	Yes	Yes
2	F	50s	Pruritic rash	-	1 year	Yes	Yes
3	F	40s	Pruritic rash	-	8	Yes	Yes
4	F	60s	Fever, Lymphadenopathy	5/5	-	Yes	Yes
5	F	40s	Pruritic rash	-	1	Yes	Yes
6	F	50s	Sore throat	-	3	Yes	Yes
7	F	60s	Fever	6/6	-	Yes	Yes
8	F	80s	Fever, Headache	NA^§^	-	NA	Yes
9	F	30s	Pruritic rash	-	3	Yes	Yes
10	F	30s	Fever, Sore throat, Arthralgia	7/ 7	-	Yes	Yes
11	F	70s	Fever	NA^¶^	-	Yes	NA^**^
12	F	30s	Pruritic rash	-	2	Yes	Yes
13	F	50s	Fever	1/ 252	-	Yes	Yes
14	F	30s	Pruritic rash	-	2	Yes	Yes
15	F	60s	Fever, Myalgia, Arthralgia	4/8	-	Yes	Yes
16	F	80s	Fever, Sore throat	16^**^	-	NA	Yes
17	F	60s	Fever, Sore throat	59/13	-	Yes	Yes
18	F	70s	Fever, Arthralgia	-/7	-	Yes	No
19	F	30s	Sore throat	-	1	Yes	Yes
20	F	70s	Fever, Skin rash	NA^*^	NA^*^	Yes	Yes
21	F	70s	Fever, Arthralgia	5/458	-	Yes	Yes

**Table 3:** Patients’ manifestations ^†^ Some patients experienced more than one initial symptom. Of eight patients (38%) who did not initially experience fever, six (29%) presented only with pruritic rash, and two (10%) presented with a sore throat alone; ^‡^In one patient, it was unclear whether the pruritic rash developed according to the record; ^§^In two patients, it was unclear whether the salmon-pink rash developed according to the record.

Initial symptoms^†^	Cases, n (%)
Fever	13/21 (62)
Pruritic rash	6/21 (29)
Sore throat	6/21 (29)
Arthralgia	4/21 (19)
Lymphadenopathy	1/21 (5)
Myalgia	1/21 (5)
Headache	1/21 (5)
Skin manifestations	Cases, n (%)
Pruritic linear streaks^‡^	19/20 (95)
Salmon-pink rash^§^	19/19 (100)

In the 13 patients, the median times from the emergence of fever to pruritic rash and salmon-pink rash were 5.5 days (interquartile range (IQR) 4.8-7.3) and eight days (IQR 7-21), respectively, with either of the cutaneous manifestations developing within 16 days (Table 4). Considering the eight out of 21 patients (38%) who did not have fever at onset, the median time from the emergence of pruritic rash to fever was 2.5 days (IQR 1.8-4.3) (Table 4). Cutaneous manifestations preceded fever in one patient, one year earlier (Case 2 in Table 2). The salmon-pink rash was always accompanied by fever.

**Table 4:** Temporal relationship between fever and skin rash ^†^ The salmon-pink rash may have developed before the days indicated in the table, as the non-pruritic nature of skin lesions could have led patients and physicians to overlook them; ^‡^ The salmon-pink rash was always accompanied by fever. IQR: interquartile range

From fever to skin rash (N=13)	Median days (IQR)
From fever to pruritic rash	5.5 (4.8-7.3)
From fever to salmon-pink rash^†^	8 (7-21)
From skin rash to fever (N=8)^‡^	Median days (IQR)
From pruritic rash to fever	2.5 (1.8-4.3)

**Figure 1:**
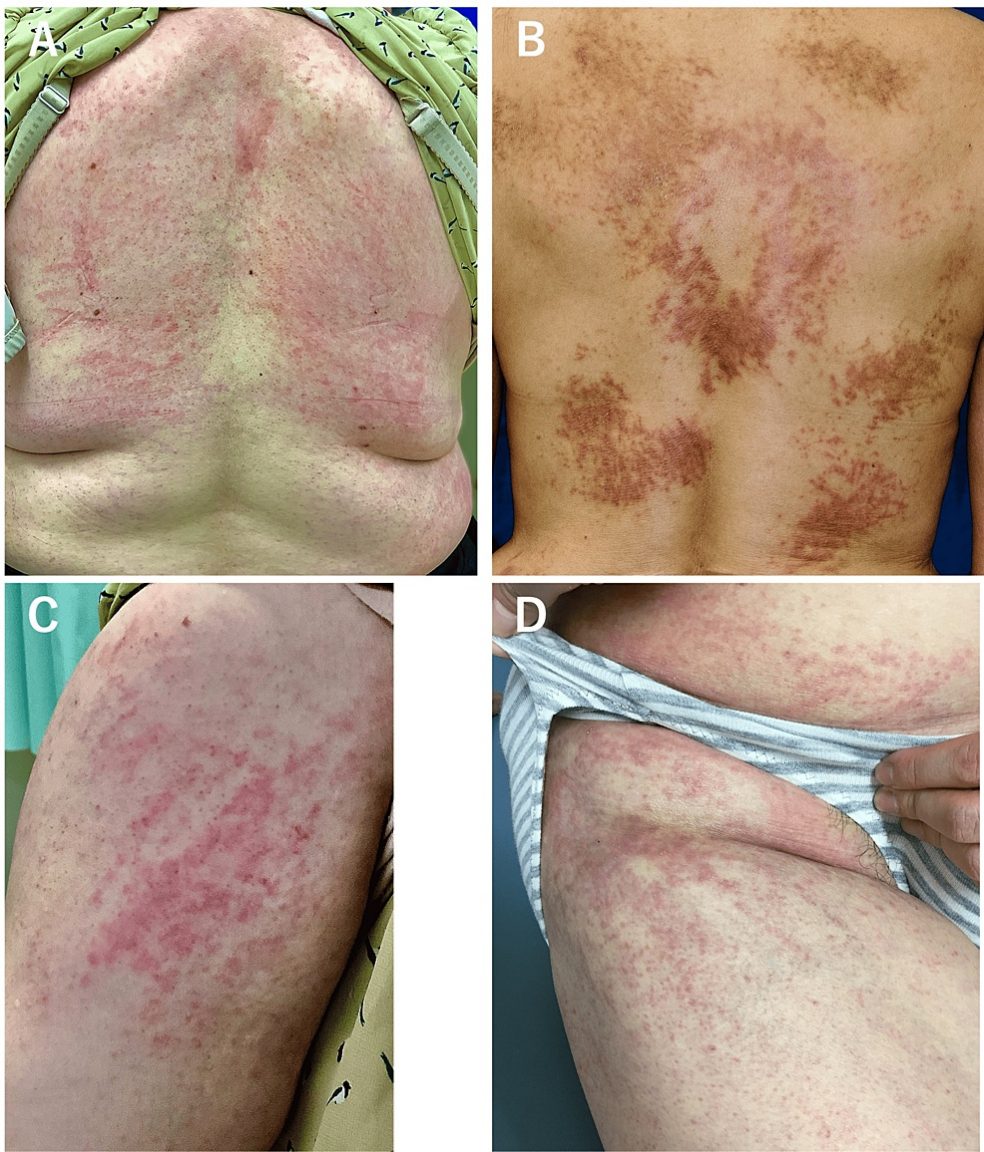
Persistent pruritic linear streaks in adult-onset Still’s disease observed on the back (A, B), brachium (C), and thigh (D) Over time, pruritic linear streaks can be accompanied by pigmentation (B). Pruritic linear streaks can be accompanied by other eruptions. For additional details, please refer to Figure 2.

**Figure 2:**
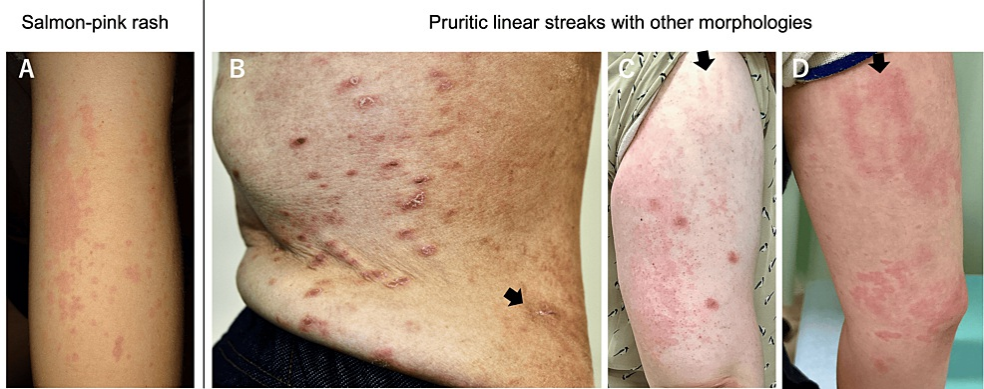
“Typical Rash”, known as the salmon-pink rash, of the Yamaguchi criteria (A) and pruritic eruptions outside the criteria including linear streaks (B-D) The “salmon-pink” macular and maculopapular nonpruritic rash erupted on the upper extremity (A). Linear streaks, accompanied by prurigo nodularis (B, C) and urticarial dermatitis (D), occurred on the trunk (B) and upper extremities (C, D). They were pruritic and persistent. Arrows indicate the linear streaks (B-D). Despite its evanescent nature, the properties of this typical rash are indistinguishable from viral or drug eruptions upon visible inspection (A). Linear streaks on the back are usually more pronounced than on the extremities (refer to Figure 1). Panels B and C depict the same patients as shown in Figure 1B and Figures 1A, 1C, respectively. Both the typical rash and pruritic eruptions may coexist in a single patient.

All 21 patients exhibited at least one of the following rashes: salmon-pink rash or pruritic linear streaks on the trunk. Nineteen patients presented a salmon-pink rash while the remaining two did not have clear records regarding this. Additionally, 19 patients displayed pruritic linear streaks on the trunk while one was without clear records about this. Some patients also exhibited pruritic linear streaks on their limbs (Figure 1). Linear streaks on the back were usually more pronounced than on the extremities. Pruritic linear streaks were accompanied by other morphologies such as prurigo nodularis and urticarial dermatitis in some patients (Figure 2). They persisted until the underlying condition improved with immunosuppressive therapy such as glucocorticoids. Linear streaks persisted regardless of fever, whereas a salmon-pink evanescent rash developed after fever. There were no specific temporal associations between the eruptions.

None of the 21 patients were correctly diagnosed at the first visit; infection was diagnosed in 14 patients (67%), an allergic condition in four (19%), drug eruption in three (14%), and urticaria in one (5%). At the referring medical institutions, skin rashes were initially diagnosed in the 21 patients as follows: drug-induced eruption in 11 (52%), viral rash in five (24%), allergic condition in two (10%), and connective tissue disease in two (10%) (Tables 5, 6).

**Table 5:** Clinical course prior to the diagnosis of AOSD ^†^ There was a possibility that the pruritic rash and salmon-pink rash had developed at a previous hospital, but the date of emergence of salmon-pink rash and whether the pruritic rash developed were unclear in the record; ^‡^ Some kind of skin rash associated with AOSD developed on day 17 (16 days after fever), but details were unavailable in the records from the previous hospital; §: The order of appearance of the skin rash and fever was unknown. Additionally, the date of emergence of the pruritic rash and salmon-pink rash was unknown. AOSD: adult-onset Still’s disease; CTD: connective tissue disease; EBV: Epstein-Barr virus; NSAID: non-steroidal anti-inflammatory drug

Case (N=21)	Initial diagnosis at first visit	Initial diagnosis for skin rash at referral	Number of medical institutions visited	Days required from initial symptoms to diagnosis (days)	Days required from pruritic rash to diagnosis (days)
1	Infection	Allergy (unknown allergen)	3	22	14
2	Allergy	Viral rash	2	1 year	1 year
3	Drug eruption (NSAIDs)	Viral rash	3	1 year	1 year
4	Infection	Drug eruption (antibiotics)	5	43	38
5	Allergy	Allergy (unknown allergen)	4	25	25
6	Allergy	CTDs	3	41	40
7	Infection	Anaphylaxis (antibiotics)	3	19	13
8	Infection	CTDs	2	41	4
9	Infection	Viral rash	3	17	17
10	Infection	Drug eruption (NSAIDs)	4	10	3
11	Infection	Drug eruption (unknown)	2	1 year	NA^†^
12	Infection or allergy	Drug eruption (antibiotics) or viral rash	4	64	64
13	Infection	Drug eruption (antibiotics)	2	252	251
14	Urticaria	Drug eruption (antibiotics)	3	12	12
15	Infection	Drug eruption (antibiotics or anticonvulsants)	4	8	4
16	Infection	Drug eruption (NSAIDs)	2	60	NA^‡^
17	Infection	Drug eruption （antihypertensives, acetaminophen or antitussives)	2	68	3
18	Infection	Unknown	2	13	-
19	Drug eruption (antibiotics)	Drug eruption (antibiotics)	1	22	21
20	Drug eruption (unknown)	Drug eruption (antibiotics)	2	4.5 months	NA^§^
21	Infection (EBV)	Viral rash (EBV)	1	458	453

**Table 6:** Initial diagnoses at first visit and referral (N=21) ^†^ One patient initially received diagnoses of “infection or allergy” and “drug eruption or viral eruption” during the first visit and referral, respectively. CTD: connective tissue disease

For symptoms at first visit^†^	Cases, n (%)
Infection	14 (67%)
Allergy	4 (19%)
Drug eruption	3 (14%)
Urticaria	1 (5%)
For skin rash at referral^†^	
Drug eruption	11 (52%)
Viral rash	5 (24%)
Allergy	2 (10%)
CTDs	2 (10%)
Anaphylaxis	1 (5%)
Unknown	1 (5%)

All patients required multiple visits to a medical institution, with a median time of 41 days (IQR 19-138) from the initial symptoms and 21 days (IQR 12-64) from the onset of pruritic rash to obtain the diagnosis of AOSD (Table 7). The patients who took a longer time (one: 4.5 months, one: 252 days, three: one year, and one: 458 days) had polycyclic patterns, with repeated flares and spontaneous remission (see Appendices).

**Table 7:** Required days and numbers of medical Institutions for diagnosis

Time to diagnosis	Median days (IQR)
From initial symptoms to diagnosis	41 (19-138)
From pruritus to diagnosis	21 (12-64)
Number of medical institutions required for diagnosis	Number (IQR)
Medical institutions visited until diagnosis	3 (2-3)

## Discussion

This study suggests that persistent pruritic erythematous linear streaks could be useful for diagnosing AOSD.

The diagnosis of AOSD is typically based on the Yamaguchi criteria. However, a pitfall in this practice is its exclusive focus on nonpruritic salmon-pink rashes as a cutaneous feature. The classification criteria for AOSD proposed by Fautrel et al. emphasize only transient erythema and maculopapular rash [4]. A pink-colored eruption is not specific to AOSD and can occur in drug eruptions and viral infections. Patients with AOSD commonly exhibit symptoms like fever and sore throat, which are also indicative of more prevalent upper respiratory tract infections. Highlighting that salmon-pink rashes are nonpruritic and painless is important; physicians often overlook the features of emergence and evanescence. Consequently, they are prone to misdiagnose AOSD as either viral infections or drug-related eruptions.

Previous studies have identified pruritic eruptions as atypical cutaneous manifestations of AOSD [2,5-24]. One case series reported persistent pruritic erythema and the typical rash in 78% and 86% of patients with AOSD, respectively [6]. This indicates that the frequency of pruritic eruptions is comparable to that of typical rashes, suggesting that it is too common to be considered atypical, as demonstrated in our study. Pruritic lesions can manifest as the initial symptoms of AOSD, preceding the onset of classic manifestations (29% of our cases) [14]. In our literature review from the PubMed database, we found quite a few reports of cases presenting with rashes consistent with persistent pruritic linear streaks as observed in our study [2,5-24]. These reports indicated that persistent pruritic linear streaks typically appear on the back and, in some patients, also on the chest, abdomen, limbs, and face (Table 8). These streaks have been described as dermatomyositis-like, erythematous and maculopapular, flagellate-like, hyperpigmented, lichenoid, persistent pruritic papules and plaques, urticarial, and other linear eruptions in different studies (Table 9). Persistent pruritic linear lesions appear as a common feature among these various skin lesions. Despite their commonality and numerous past reports, pruritic eruptions are still reported as "atypical" or "rare," possibly because common findings associated with pruritic eruptions had not been mentioned until now and the Yamaguchi criteria remain unmodified.

**Table 8:** Descriptions of persistent pruritic linear streaks in the literature

Descriptions	Site	Reference
Dermatomyositis-like lesions, Erythematous maculopapular eruption resembling Keobner phenomenon	Back, Leg	Qiao et al. [5]
Dermatomyositis-like pursistent pruritic eruptions, Dusky red linear lesions, Erythematous linear lesions, Linear pigmented lesions, Lichenoid papules of persistent pruritic eruptions, Urticarial and lichenoid papules	Back, Abdomen	Lee et al. [6]
Erythematous linear plaques	Back	Awoyemi et al. [7]
Erythematous to brownish maculopapular rash in rippled pattern	Back, Chest, Abdomen	Khullar et al. [8]
Erythematous, urticarial eruption with linear streaks, Linear urticarial wheals	Back, Buttocks, Abdomen, Limb, Face	Prendiville et al. [9]
Erythrematous maculopapular pruritic rash	Back, Chest, Shoulder	Shar et al. [10]
Erythematous papules with with linear configuration, Linear pigmented streaks, Persistent pigmented plaques with linear configuration	Back, Buttocks, Chest, Abdomen, Limb	Yamamoto [11]
﻿Fixed, pruritic, mildly scaly, pink to erythematous, blanchable plaques	Back, Neck	Fortna et al. [12]
Flagellate dermatitis	Back	Riyaz et al. [13]
Flagellate erythema	Back	Ciliberto et al. [2]
Flagellate erythema-type appearance	Back, Chest, Limb	Narváez Garcia et al. [14]
Linear erythematous streaks (Flagellate erythema)	Trunk, Back, Stomach	Toujani et al. [15]
Whiplash-like erythematous streaks (Flagellate erythema)	Back	Bhatia et al. [16]
Hyperpigmented linear streaks, Hyperpigmented macules and papules with a linear rippled pattern	Back, Leg	Santa et al. [17]
Hyperpigmented papules and plaques with fine overlying scale with secondary linear excoriations	Back	Whittington et al. [18]
﻿Linear pigmented streaks Linear erythema	Back	Kikuchi et al. [19]
Maculopapular, scaly rash in a linear pattern	Back, Buttocks, Abdomen, Limb, Face	Wolgamot et al. [20]
Persistent oedematous erythema with linear configurations	Back, Abdomen	Liu et al. [21]
Persistent pruritic lesions with bizzare linear lesions, Persistent pruritic lichenoid lesions with bizzare linear array	Back, Neck	Lee et al. [22]
Pruritic edematous erythema and persistent papules and plaques with prominent linear pigmentation	Back, Chest	Suzuki et al. [23]
Pruritic, erythematous plaques and dark-reddish papules	Back, Abdomen	Yoshifuku et al. [24]

**Table 9:** Subcategories of persistent pruritic linear streaks in the literature

Subcategories	Descriptions	Reference
Dermatomyositis-like	Dermatomyositis-like lesions	Qiaoet al. [5]
	Dermatomyositis-like persistent pruritic eruptions	Lee et al. [6]
Erythematous and maculopapular	Erythematous maculopapular eruption resembling Koebner phenomenon	Qiaoet al. [5]
	Erythematous to brownish maculopapular rash in rippled pattern	Khullar et al. [8]
	Erythematous maculopapular pruritic rash	Shar et al. [10]
	Erythematous papules with linear configuration	Yamamoto [11]
	Erythematous linear plaques	Awoyemi et al. [7]
	Maculopapular, scaly rash in a linear pattern	Wolgamot et al. [20]
	Fixed, pruritic, mildly scaly, pink to erythematous, blanchable plaques	Fortna et al. [12]
Flagellate-like	Flagellate dermatitis	Riyaz et al. [13]
	Flagellate erythema	Ciliberto et al. [2]
	Flagellate erythema-type appearance	Narváez Garciaet al. [14]
	Linear erythematous streaks (Flagellate erythema)	Toujani [15]
	Whiplash-like erythematous streaks (Flagellate erythema)	Bhatia et al. [16]
Hyperpigmented	Hyperpigmented linear streaks	Santa et al. [17]
	Hyperpigmented macules and papules with a linear rippled pattern	Santa et al. [17]
	Hyperpigmented papules and plaques with fine overlying scale with secondary linear excoriations	Whittington et al. [18]
	Linear pigmented lesions	Lee et al. [6]
	Linear pigmented streaks	Kikuchi et al. [19]
Lichenoid	Lichenoid papules of persistent pruritic eruptions	Lee et al. [6]
	Persistent pruritic lichenoid lesions with bizarre linear array	Lee et al. [22]
Persistent pruritic papules and plaques	Persistent pruritic lesions with bizarre linear lesions	Lee et al. [22]
	Pruritic, erythematous plaques and dark-reddish papules	Yoshifuku et al. [24]
	Pruritic edematous erythema and persistent papules and plaques with prominent linear pigmentation	Suzuki et al. [23]
Urticarial	Erythematous, urticarial eruption with linear streaks	Prendiville et al. [9]
	Linear urticarial wheals	Prendiville et al. [9]
	Urticarial and lichenoid papules	Lee et al. [6]
Other linear eruptions	Dusky red linear lesions	Lee et al. [6]
	Erythematous linear lesions	Lee et al. [6]
	Linear erythema	Kikuchi et al. [19]
	Persistent edematous erythema with linear configurations	Liu et al. [21]

Pruritic linear streaks in AOSD may be provoked by scratching, a response known as the Koebner phenomenon [2,5,6,12,17,19,22]. These streaks are usually erythematous but can become pigmented in the chronic phase [6,17-19]. Additionally, they can be accompanied by other pruritic eruptions such as prurigo nodularis, urticarial dermatitis, and crusted lichenoid papules [6,9,22]. The severity of pruritic linear streaks varies among patients, ranging from widespread skin rashes to localized areas. Pruritic linear streaks in AOSD may mimic dermatographism and flagellate erythema. Dermatographism, an urticarial eruption provoked by physical stimuli like scratching, typically begins to fade within 30 minutes, with H1 antihistamines effectively controlling symptoms [25,26]. In patients with AOSD, although scratching may induce linear streaks, they persist until the underlying condition improves with immunosuppressive therapy such as glucocorticoids, and antihistamines prove ineffective in managing pruritus and eruptions, as indicated in our study (See Appendices).

Flagellate erythema, characterized by “whip-like” linear streaks on the trunk, is associated with chemotherapy, particularly bleomycin, and undercooked mushroom ingestion, specifically Shiitake mushrooms. AOSD and dermatomyositis can cause similar eruptions [5,6]. Dermatomyositis typically presents with additional skin manifestations such as nailfold capillary abnormalities, Gottron’s sign/papules, and heliotrope rash. We define persistent pruritic linear streaks of AOSD as follows: (i) persistent until the condition of AOSD improves, (ii) pruritic, (iii) appearing in a linear configuration, possibly caused by scratching (Koebner phenomenon), and (iv) encompassing the various descriptions previously reported, such as dermatomyositis-like, erythematous and maculopapular, flagellate-like, hyperpigmented, lichenoid, persistent pruritic papules and plaques, and urticarial eruptions. The combination of persistent pruritic linear streaks and an evanescent rash in the context of spiking fever, sore throat, and arthralgia strongly suggest AOSD [22], as indicated in our study.

Histological analysis may assist in differential diagnosis in some cases. The features of flagellate erythema align with common patterns of exanthematous/morbilliform drug reactions, characterized by epidermal spongiosis, interface dermatitis, and a dermal perivascular lymphocytic infiltrate with eosinophils [27]. In dermatomyositis, the histological features include vacuolar interface dermatitis with a dermal perivascular lymphocytic infiltrate. The histology of AOSD is characterized by dyskeratotic keratinocytes with a dermal perivascular neutrophil infiltrate along with lymphocytes [22]. However, the histological findings alone are not sufficient evidence for a definitive diagnosis. In most cases, the correct diagnosis can be made through history-taking, physical examination, and laboratory examination, and performing a skin biopsy is not mandatory.

The early diagnosis of AOSD remains a significant challenge in clinical practice due to the nonspecific nature of its manifestations and the limitations of current diagnostic criteria, particularly the Yamaguchi criteria, which do not include pruritic rashes [1]. When patients present with pruritic rash, physicians may not consider AOSD in the different diagnoses. In our study, despite a considerable number of patients visiting general practitioners or private practice dermatologists with pruritus and fever, AOSD was rarely considered. Furthermore, strict adherence to the Yamaguchi criteria can hinder diagnostic consensus among physicians when patients exhibit pruritic rashes, potentially contributing to diagnostic delays and disease exacerbation, such as hemophagocytic lymphohistiocytosis, a major cause of mortality in patients with AOSD, as 19% of the patients experienced hemophagocytic lymphohistiocytosis in our study (See Appendices) [28].

A nationwide survey in Japan involving 169 patients with AOSD indicates that the typical rash outlined in the Yamaguchi criteria may be less frequent than previously thought (86.7%), with only 62.2% of patients with AOSD exhibiting it [29]. Data from this survey were collected through questionnaires, and the diagnosis of AOSD was based on physicians’ judgment rather than the Yamaguchi criteria. Although the study population was exclusively Japanese, which limits the generalizability of the findings to other ethnicities and geographic regions, this survey suggests a potential gap between the criteria proposed by Yamaguchi et al. [1] and real-world diagnoses as our study indicates unmet medical needs regarding the diagnostic process of AOSD.

Our study aimed to identify additional clues for an early diagnosis beyond the existing criteria. Therefore, we extracted data of patients with AOSD from clinical records that strictly met the Yamaguchi criteria in accordance with the phrase in the footnote stating, “all criteria are applicable only in the absence of other clinical explanations” to minimize subjectivity (refer to the footnote in Table 1). As a result, all 19 patients, except two without clear documentation, exhibited a salmon-pink evanescent rash. However, it is noteworthy that two cases in our study (Nos. 8 and 16 in Table 2) were diagnosed with AOSD without a detected salmon-pink rash; instead, the diagnosis was supported by the presence of pruritic linear streaks (see Appendices). Thus, future research should investigate the occurrence of AOSD where pruritic linear streaks are present without a salmon-pink rash.

The limitations of this study include incomplete medical records due to its retrospective design; the assessment of skin lesions may be influenced by additional clinical information. Additionally, this was a pilot study, and enrolling patients from two community hospitals may not have been sufficient to yield conclusive evidence. Lastly, all patients were Japanese. Therefore, our data may not be generalizable to other ethnicities and geographical regions.

## Conclusions

Our findings highlight the diagnostic value of persistent pruritic linear streaks in AOSD. Incorporating these eruptions into classification criteria may facilitate earlier diagnosis of AOSD and improve patient outcomes. We propose that enhancing the classification criteria for AOSD may be beneficial in several ways: enabling earlier diagnosis, potentially improving disease prognosis, gaining a more accurate understanding of epidemiology, and reevaluating the clinical spectrum of AOSD. Further research is needed to validate our findings and explore the implications of expanding the diagnostic criteria for AOSD.

## Appendices

**Table 10:** Timelines of symptoms and diagnoses ^†^ Unless otherwise specified, the fever and salmon-pink rash continued to appear and disappear, while the pruritic rash persisted until the underlying condition improved; ^‡^All cutaneous manifestations were described in the clinical records by dermatologists, rheumatologists, or general physicians at the time of physical examination and/or diagnosis. Photographs were preserved in 12/21 cases (57%), serving as a means of reconfirming the manifestations. d: day; w: week; m: month; y: year; GC: glucocorticoid; AOSD: adult-onset Still’s disease; ER: emergency room; UTI: urinary tract infection; LN: lymph node; CTD: connective tissue disease; CVA/AMPC: clavulanic acid/amoxicillin; iv: intravenous; ICS: inhaled corticosteroids; HLH: hemophagocytic lymphohistiocytosis; SVT/ABPC: sulbactam/ampicillin; EBV: Epstein-Barr virus.

Case	Timeline of Symptoms and Diagnoses^†‡^
1	d1: Developed fever, sore throat, and arthralgia. d9: Experienced a pruritic rash. d10: Visited a general practitioner, who prescribed clarithromycin for an "infection." Later, visited a private practice dermatologist who prescribed an antihistamine and topical GCs for "allergy." d12: Referred to our hospital. "Allergic" reaction was suspected by a general physician. d16: Admitted to our hospital with suspicion of “rheumatic disorders.” d22: Salmon-pink rash and pruritic linear streaks with Koebner phenomenon were detected. Diagnosed as AOSD by a rheumatologist in consultation with a dermatologist.
2	Experienced a pruritic rash, which was spontaneously improved in 3m. 1y later: Developed fever, pruritic rash, and arthralgia. Visited a private practice dermatologist who prescribed oral and topical GCs for "allergy." 2d later, visited the ER at our hospital with a worsened rash. Diagnosed as “viral rash." 15d later, salmon-pink rash and pruritic linear streaks with Koebner phenomenon detected. Diagnosed as AOSD by a rheumatologist in consultation with a dermatologist.
3	Experienced a pruritic rash. 8d later: Developed fever of unknown origin which improved spontaneously in two weeks. One year later: Experienced a pruritic rash again. 4d later, developed sore throat. Visited a private practice dermatologist who prescribed oral GCs for "drug eruption." 3d later, visited another private practice dermatologist, who again prescribed oral GCs for "drug eruption." 1d later, visited the ER at our hospital with fever. Diagnosed as "viral rash." 1d later, again visited the ER and was prescribed cefalexin for "UTI." 8d later, visited a general physician at our hospital. An “infection” was suspected. 10d later, salmon-pink rash & pruritic linear streaks with Koebner phenomenon were detected. Diagnosed as AOSD by a rheumatologist.
4	d1: Developed fever and lymphadenopathy. Visited a general practitioner, who prescribed cefalexin for an "infection." d4: Experienced worsening pain in LNs. Referred to an otolaryngologist at a community hospital who prescribed levofloxacin for an “infection.” d6: Developed a pruritic rash and salmon-pink evanescent rash, sore throat, cough, and arthralgia. Visited a private practice dermatologist who prescribed an antihistamine for "allergy." d11: Referred to a general physician at a university hospital. "Drug eruption" was suspected. d18: Further referred to a general physician at our hospital. d25: Oral GCs were started as treatment for a "severe drug eruption." d36: Visited a dermatologist at our hospital. “Dermatomyositis” was suspected. d43: Consulted a rheumatologist at our hospital and diagnosed as AOSD.
5	d1: Experienced a pruritic rash. d2: Developed fever. Visited a general practitioner, who prescribed oral GC and an antihistamine for "allergy." d5: Returned to the practitioner who prescribed an additional antihistamine. d14: Visited a private practice dermatologist who prescribed an antihistamine for "allergy." d15: Revisited the dermatologist with arthralgia and myalgia. Oral GCs were prescribed. Subsequently, visited the ER at a community hospital where levofloxacin was prescribed for "UTI." d17: Consulted a general physician at the community hospital and the course of levofloxacin was continued. d18: Developed arthralgia. d19: Called for an ambulance due to general weakness and admitted to our hospital. "Allergic" reaction was suspected. d25: Salmon-pink rash and pruritic linear streaks with Koebner phenomenon were detected. Diagnosed as AOSD by a rheumatologist in consultation with a dermatologist.
6	d1: Developed sore throat. d2: Experienced a pruritic rash and arthralgia. d3: Developed salmon-pink evanescent rash. Visited a general practitioner who prescribed an antihistamine for "allergy". d4: Developed fever. Visited a private practice dermatologist who prescribed oral GCs. d5: Revisit the practitioner and loxoprofen was prescribed. d10: Transferred to our hospital by ambulance due to severe arthralgia. "CTDs" were suspected. d12: Consulted a general physician at our hospital. A suspicion of "CTDs" persisted. d21: Referred to a dermatologist at a university hospital who prescribed oral GCs. d41: Diagnosed as AOSD by a rheumatologist at our hospital.
7	d1: Developed fever, sore throat, and arthralgia. Visited a general practitioner who prescribed clarithromycin for an "infection." d5: Consulted a pulmonologist at a community hospital, where cefcapene pivoxil was prescribed. Subsequently, transferred by ambulance due to hypotension and admitted to our hospital with suspicion of "anaphylaxis." d7: Experienced a pruritic rash and salmon-pink rash. d19: Diagnosed as AOSD by a rheumatologist in consultation with a dermatologist.
8	d1: Developed fever and headache. Visited a general practitioner who prescribed loxoprofen with suspicion of "COVID-19." d31: Revisited the practitioner and fosfomycin was prescribed for "an infection." d38: Referred to a general physician at our hospital. Pruritic linear streaks were detected and suspicion of "CTDs" arose. d41: Diagnosed as AOSD by a rheumatologist.
9	d1: Experienced a pruritic rash. d3: Visited a private practice dermatologist who prescribed an antihistamine for "allergy” or a potential “infection." d4: Developed fever. d7: Visited a general practitioner who prescribed loxoprofen. d10: Visited the ER at our hospital due to severe pruritus. "Viral rash" was suspected. d13: Consulted a general physician at our hospital. "CTDs" were suspected. d16: Developed sore throat and arthralgia. d17: Salmon-pink rash and pruritic linear streaks with Koebner phenomenon were detected. Diagnosed as AOSD by a rheumatologist.
10	d1: Developed fever, sore throat, and arthralgia. d2: Visited a general physician, who prescribed cephem antibiotics and loxoprofen for an “infection." d4: Consulted another general physician and CVA/AMPC was prescribed. d6: Referred to a pulmonologist at a community hospital, transferred to our hospital due to pericarditis and myocarditis, and admitted. d8: Salmon-pink rash and pruritic linear streaks with Koebner phenomenon were detected by a general physician. "Drug eruption" was suspected. d10: Diagnosed as AOSD by a rheumatologist.
11	Developed fever and was admitted to a university hospital with suspicion of an “infection." Fever improved spontaneously in 5m. 1y later: Developed fever and myalgia. Referred to a general physician at our hospital. IV ceftriaxone administered for "infection." 2d later, admitted to our hospital. 3d later, developed sore throat and salmon-pink rash. "Drug eruption" was suspected. 5d later, diagnosed as AOSD by a general physician.
12	d1: Experienced a pruritic rash. Visited a general practitioner who prescribed an antihistamine and loxoprofen for an "infection” or “allergy." d3: Visited a private practice dermatologist who prescribed an antihistamine for "allergy." Soon after, developed fever, sore throat, and cough. d26: Revisited the general practitioner, and ICS and montelukast were prescribed for "cough variant asthma." d31: Consulted another general practitioner who prescribed clarithromycin for an "infection." d50: Visited the ER at our hospital. "Infectious monocytosis,” “drug eruption,” and “viral rash" were suspected. d63: Admitted to our hospital due to persistent fever and skin rash. d64: Salmon-pink rash and pruritic linear streaks with Koebner phenomenon were detected. Diagnosed as AOSD by a rheumatologist.
13	d1: Developed fever. d2: Experienced a pruritic rash with Koebner phenomenon. d5: Developed sore throat. Visited a dermatologist at our hospital and diagnosed as "toxicoderma" due to pharyngitis. d14: The symptoms improved. d222: Developed fever and sore throat. d224: Visited a general practitioner who prescribed azithromycin for an “infection." Subsequently visited a private practice dermatologist who prescribed an antihistamine. Soon after, experienced a pruritic rash with Koebner phenomenon. d225: Developed arthralgia. d230: Revisited the practitioner. IV ceftriaxone was administered. d231: Revisit the practitioner. IV Hydrocortisone administered for "drug eruption." Revisited the dermatologist; oral and topical GCs were prescribed. d232: Referred to a dermatologist at our hospital. The course of oral GCs continued. d236: Admitted to our hospital due to fever, arthralgia, and pruritic linear streaks with Koebner phenomenon. d252: Salmon-pink rash was detected. Diagnosed as AOSD by a rheumatologist with consent of a dermatologist.
14	D1: Experienced a pruritic rash. d2: Visited a private practice dermatologist and diagnosed as “urticaria." However, the patient doubted the diagnosis and consulted a general practitioner who prescribed garenoxacin for an "infection." d3: Developed fever. d7: Developed sore throat. d12: Admitted to our hospital due to fever and a pruritic rash. A dermatologist suspected "drug eruption," but was complicated by HLH. Salmon-pink rash and pruritic linear streaks with Koebner phenomenon were detected. Diagnosed as HLH due to AOSD by a rheumatologist (with the later consent of a dermatologist).
15	d1: Developed fever, myalgia, and arthralgia. d4: Visited a general practitioner and diagnosed as "UTI" for which cefcapene pivoxil and loxoprofen were prescribed. d5: Experienced a pruritic rash. d7: Consulted another general practitioner who suspected "drug eruption." Subsequently referred and admitted to our hospital. d8: Complicated by HLH. Salmon-pink rash & pruritic linear streaks with Koebner phenomenon were detected. Diagnosed as HLH due to AOSD by a rheumatologist in consultation with a dermatologist.
16	d1: Developed fever and sore throat. d8: Admitted to a community hospital, where a physician detected arthritis. IV SBT/ABPC was administered for "pharyngitis." d15: Loxoprofen was prescribed. d17: Developed a rash (details unknown). Diagnosed with "drug eruption" by a physician. IV GCs were started. d32: GC treatment shifted to oral GCs. d60: Referred to our hospital and diagnosed as AOSD by a rheumatologist. Pruritic linear streaks detected at a relapse, although it was unclear whether salmon-pink rashes had developed at the previous hospital.
17	d1: Developed fever and sore throat. d3: Visited a general practitioner who prescribed levofloxacin for "pharyngitis." d6: Referred to our hospital, where a general physician supported a diagnosis of "pharyngitis." d14: Experienced a non-pruritic salmon-pink rash. Later, fever, sore throat, and rash improved spontaneously. d60: Experienced a pruritic rash and sore throat. d66: Admitted to our hospital due to fever, pruritic rash, and arthralgia. d67: Pruritic linear streaks with Koebner phenomenon were detected. A general physician suspected "drug eruption." d68: Complicated by HLH. Salmon-pink rash was detected. Diagnosed by a rheumatologist in consultation with a dermatologist.
18	d1: Developed fever and arthralgia. d2: Visited a general practitioner, who prescribed prulifloxacin and ibuprofen for an "infection." d3: IV SBT/ABPC was administered at the practitioner’s office. d8: Admitted to our hospital due to fever, arthralgia, and a salmon-pink rash of "unknown causes." d13: Diagnosed as AOSD by a rheumatologist.
19	d1: Developed sore throat and took cefditoren pivoxil on hand. d2: Experienced a pruritic rash. Visited a general practitioner who prescribed an antihistamine and topical GCs for "drug eruption." Soon after, developed fever and arthralgia. d9: Revisited the practitioner; cefditoren pivoxil was prescribed for an “infection.” d17: Admitted to our hospital due to fever, pruritic rash, and arthralgia. Pruritic linear streaks were detected. A general physician suspected "drug eruption." d22: Salmon-pink rash was detected. Diagnosed as AOSD by a rheumatologist.
20	Developed fever and skin rash (details unknown). Visited a private practice dermatologist and diagnosed as "drug eruption." m1.5: Admitted to a mental hospital due to severe depression. m2: Developed fever, arthritis, and skin rash (details unknown) which improved with oral GCs. m3: Developed fever and skin rash again. m3.5: Transferred from a mental hospital to our hospital due to fever and skin rash. Pruritic linear streaks were detected, and a general physician suspected "drug eruption." m4.5: Salmon-pink rash was detected. Diagnosed as AOSD with a tendency toward HLH by a rheumatologist.
21	d1: Developed fever and arthralgia. d6: Experienced a pruritic rash. d7: Visited a general practitioner who suspected infectious monocytosis due to EBV. d10: Referred to our hospital, where a general physician suspected "viral rash." Fever and rash improved spontaneously thereafter. d32: Developed fever again. d39: Fever improved spontaneously. d448: Developed fever again. d458: Salmon-pink rash and pruritic linear streaks with Koebner phenomenon were detected. Diagnosed as AOSD by a rheumatologist.
